# Polymer Materials for Drug Delivery and Tissue Engineering

**DOI:** 10.3390/polym15143103

**Published:** 2023-07-21

**Authors:** Ariana Hudiță, Bianca Gălățeanu

**Affiliations:** Department of Biochemistry and Molecular Biology, University of Bucharest, 91-95 Splaiul Independentei Street, 050095 Bucharest, Romania; bianca.galateanu@bio.unibuc.ro

In recent years, the biomedical engineering field has seen remarkable advancements, focusing mainly on developing novel solutions for enhancing tissue regeneration or improving therapeutic outcomes. One of the key components of the latest developments is polymers (natural, synthetic, or blended formulations), due to their indisputable wide range of properties and functionalities that transform them into ideal materials for designing drug delivery systems or scaffolds for tissue engineering.

The Special Issue “Polymer Materials for Drug Delivery and Tissue Engineering” gathers a multidisciplinary collection of articles that merge the knowledge and expertise of worldwide researchers that present their latest research in the field of polymer-based materials. This Special Issue brings together articles that report original research on the design and synthesis of novel drug delivery systems and scaffolds or highlight insights into polymer interaction [1,2,3,4,5]. To improve the current knowledge and open up new perspectives in the use of biopolymer blends, Wilharm et al. [6] characterized the interaction of collagen and elastin fibers during polymerization and revealed that elastin is incorporated homogeneously into the collagen fibers. The results contribute significantly to designing elastin-based biomaterials with or without actuatoric applications. In another study [7], a bioactive scaffold obtained based on demineralized human spongiosa Lyoplast was tested to reveal its microstructure and biochemical properties. The reported findings validate the biopolymer for its further use in developing scaffolds for articular hyaline cartilage tissue engineering, as the material preserves the typical hierarchical porous structure and the presence of collagen and other extracellular matrix proteins, with no cytotoxic-mediated effect in vitro on chondroblasts. Moreover, Laomeephol et al. [8] revealed the advantages of blending *Bombyx mori* silk fibroin (SF) and recombinant spider silk protein eADF4(C16) into a hydrogel that showed superior mechanical and biological performance when compared with the characteristics of individual materials.

Furthermore, this Special Issue reveals other biomedical applications of polymers such as the development of organ-on-a-chip platforms. With a focus on the urology field, Galateanu et al. [9] presented an overview of the use of polymers in developing microfluidic devices for creating physiological organ biomimetic systems for the kidney, bladder, and prostate using lithography, or bioprinting, representing valuable tools for understanding disease mechanisms or developing more effective drugs.

For tissue engineering purposes, polymers are widely used for the design and fabrication of biomimetic scaffolds that can substitute the defective tissue while maintaining a suitable environment to promote healing [10]. Depending on the intended application, a polymer-based scaffold can be designed to mimic the natural extracellular matrix (ECM) and therefore provide mechanical support for cellular adhesion, proliferation, and differentiation in a 3D manner that resembles the complex architecture of the targeted defective tissue. Scaffolds can be easily tuned depending on the requirements imposed by the intended applications in terms of mechanical properties, degradation rates, and surface characteristics, this versatility being the main reason why the same polymer can be used for the development of a variety of scaffolds that meet different tissue engineering applications. Moreover, the scaffold can be tailored to encapsulate and act as a reservoir for soluble bioactive molecules that can be released in a controlled manner or as a response to cues provided by the biological microenvironment. For example, polycaprolactone (PCL) was employed as a starting material for the development of multiple scaffolds that respond to different potential tissue restorative applications [11,12,13]. Busuioc et al. [11] manufactured bioactive and bioresorbable composite materials based on PCL and calcium magnesium silicate powders through an electrospinning technique to aid and sustain bone tissue regeneration. PCL-based scaffolds were also developed to feature antibacterial properties, as infections are frequently associated with tissue injuries. An oxidized regenerated cellulose/PCL bilayered composite (ORC/PCL) [12] incorporating different concentrations of cefazolin was screened to select the best formulation that can act as a synthetic dural substitute, with proper antibacterial activity. Despite the observations that drug incorporation triggers mechanical and physical changes in comparison with pristine scaffolds, the ORC/PCL composite loaded with 2.5 g cefazolin was selected as a promising formulation resembling the microstructure and physical and mechanical properties of the drug-unloaded composite while featuring antibacterial activity sustained for up to 4 days. Khunova et al. [13] also proposed the use of PCL for designing a bioactive scaffold that can act as a potential substrate to prevent bacterial infections associated with wound healing. Using electrospinning, PCL nanofibers reinforced by halloysite nanotubes (HNTs) loaded with erythromycin were manufactured, as a strategy to overcome the low solubility of the drug in aqueous solutions. The addition of HNTs in the PCL nanofibers improved the mechanical properties of the final scaffold, which presented a superior Young modulus and tensile strength and a strong antibacterial effect on both Gram-negative (*Escherichia coli*) and Gram-positive (*Staphylococcus aureus*) bacteria.

For wound healing applications, Al. Arjan and collaborators [14] proposed a pH-sensitive dressing prepared by blending bacterial cellulose (BC) with polyvinyl alcohol (PVA) and graphene oxide (GO), which was studied as a potential drug delivery platform after curcumin loading. The GO content impacts the physical–mechanical performance of the composite hydrogel in terms of the biodegradation rate, and mechanical and hydrophilicity properties. In vitro studies on Gram-positive and Gram-negative bacteria, as well as on cancer cells, revealed a dual role of the pH-responsive PVA/BC-*f*-GO dressing material that exhibits both antimicrobial and anticancer activities.

As in tissue engineering applications, the biocompatibility and biodegradability of polymers are essential features that promote their use for drug delivery system development, such as nanoparticles, micelles, and hydrogels [15]. Drug delivery systems improve drug solubility, protect the drug load from degradation, decrease the adverse effects associated with free-drug administration, and can be easily tuned to feature a controlled release of the drug suitable for the desired application. A strategy for modulating the release of the loaded drugs is the design of stimuli-responsive drug delivery systems. Such an example is the temperature- and pH-responsive poly(N-isopropyl acrylamide)-co-poly(acrylamide) (PNIPAM-co-PAAm) drug delivery system fabricated by Thirupathi and collaborators [16]. To validate the drug delivery behavior, curcumin was employed as a model drug, with the results showing a nearly complete release of the cargo in the presence of combined pH and temperature.

Moreover, especially for drug delivery systems developed for cancer treatment, functionalization strategies can be employed to direct nanoshuttles to tumor cells, thus increasing the therapeutic efficacy of the drug cargo while reducing toxicity [15]. For colorectal cancer management, Ullah et al. [17] designed chitosan nanoparticles loaded with 5-fluorouracil (5-FU) and decorated with folic acid (FA) to improve folate receptor affinity. Their results revealed the enhanced cytotoxicity of the 5-FU-FA chitosan nanoparticles compared to free 5-FU and non-functionalized 5-FU chitosan nanoparticles in Caco2 tumor cell cultures. However, functionalization strategies are not mandatory for developing effective nanoshuttles for anticancer drug delivery, since nanosized drug delivery systems accumulate preferentially in tumor cells, due to the abnormalities of the tumor vasculature. A nanosystem based on *Bombyx mori* silk sericin was successfully developed for doxorubicin delivery and induced cytotoxicity and genotoxicity in MCF-7 tumor cells selected as an in vitro model for breast cancer [18].

In conclusion, polymers are valuable tools for the development of drug delivery systems and biomimetic scaffolds due to their tailorable design, versatility, attractive physiochemical properties, and excellent biocompatibility. With ongoing advancements in polymer science and personalized medicine, polymers hold great promise in revolutionizing the current landscape of the biomedical field, providing a real opportunity for designing personalized biomedical products that can be easily adjusted to meet patients’ needs.

## References

[1] Rizg W.Y., Hosny K.M., Eshmawi B.A., Alamoudi A.J., Safhi A.Y., Murshid S.S., Sabei F.Y., Al Fatease A. (2022). Tailoring of Geranium Oil-Based Nanoemulsion Loaded with Pravastatin as a Nanoplatform for Wound Healing. Polymers.

[2] Aljubailah A., Alqahtani S.M., Al-Garni T.S., Saeed W.S., Semlali A., Aouak T. (2022). Naproxen-Loaded Poly (2-Hydroxyalkyl Methacrylates): Preparation and Drug Release Dynamics. Polymers.

[3] Zahariev N., Marudova M., Milenkova S., Uzunova Y., Pilicheva B. (2021). Casein Micelles as Nanocarriers for Benzydamine Delivery. Polymers.

[4] Latif M.S., Azad A.K., Nawaz A., Rashid S.A., Rahman M.H., Al Omar S.Y., Bungau S.G., Aleya L., Abdel-Daim M.M. (2021). Ethyl Cellulose and Hydroxypropyl Methyl Cellulose Blended Methotrexate-Loaded Transdermal Patches: In Vitro and Ex Vivo. Polymers.

[5] Vigneswari S., Gurusamy T.P., Khairul W.M., HPS A.K., Ramakrishna S., Amirul A.-A.A. (2021). Surface Characterization and Physiochemical Evaluation of P(3HB- Co-4HB)-Collagen Peptide Scaffolds with Silver Sulfadiazine as Antimicrobial Agent for Potential Infection-Resistance Biomaterial. Polymers.

[6] Wilharm N., Fischer T., Hayn A., Mayr S.G. (2022). Structural Breakdown of Collagen Type I Elastin Blend Polymerization. Polymers.

[7] Tsiklin I.L., Pugachev E.I., Kolsanov A.V., Timchenko E.V., Boltovskaya V.V., Timchenko P.E., Volova L.T. (2022). Biopolymer Material from Human Spongiosa for Regenerative Medicine Application. Polymers.

[8] Laomeephol C., Vasuratna A., Ratanavaraporn J., Kanokpanont S., Luckanagul J.A., Humenik M., Scheibel T., Damrongsakkul S. (2021). Impacts of Blended Bombyx Mori Silk Fibroin and Recombinant Spider Silk Fibroin Hydrogels on Cell Growth. Polymers.

[9] Galateanu B., Hudita A., Biru E.I., Iovu H., Zaharia C., Simsensohn E., Costache M., Petca R.-C., Jinga V. (2022). Applications of Polymers for Organ-on-Chip Technology in Urology. Polymers.

[10] Socci M.C., Rodríguez G., Oliva E., Fushimi S., Takabatake K., Nagatsuka H., Felice C.J., Rodríguez A.P. (2023). Polymeric Materials, Advances and Applications in Tissue Engineering: A Review. Bioengineering.

[11] Busuioc C., Alecu A.-E., Costea C.-C., Beregoi M., Bacalum M., Raileanu M., Jinga S.-I., Deleanu I.-M. (2022). Composite Fibers Based on Polycaprolactone and Calcium Magnesium Silicate Powders for Tissue Engineering Applications. Polymers.

[12] Sanpakitwattana A., Suvannapruk W., Chumnanvej S., Hemstapat R., Suwanprateeb J. (2022). Cefazolin Loaded Oxidized Regenerated Cellulose/Polycaprolactone Bilayered Composite for Use as Potential Antibacterial Dural Substitute. Polymers.

[13] Khunová V., Kováčová M., Olejniková P., Ondreáš F., Špitalský Z., Ghosal K., Berkeš D. (2022). Antibacterial Electrospun Polycaprolactone Nanofibers Reinforced by Halloysite Nanotubes for Tissue Engineering. Polymers.

[14] Al-Arjan W.S., Khan M.U.A., Almutairi H.H., Alharbi S.M., Razak S.I.A. (2022). PH-Responsive PVA/BC-f-GO Dressing Materials for Burn and Chronic Wound Healing with Curcumin Release Kinetics. Polymers.

[15] Ginghină O., Hudiță A., Zaharia C., Tsatsakis A., Mezhuev Y., Costache M., Gălățeanu B. (2021). Current Landscape in Organic Nanosized Materials Advances for Improved Management of Colorectal Cancer Patients. Materials.

[16] Thirupathi K., Phan T.T.V., Santhamoorthy M., Ramkumar V., Kim S.-C. (2022). PH and Thermoresponsive PNIPAm-Co-Polyacrylamide Hydrogel for Dual Stimuli-Responsive Controlled Drug Delivery. Polymers.

[17] Ullah S., Azad A.K., Nawaz A., Shah K.U., Iqbal M., Albadrani G.M., Al-Joufi F.A., Sayed A.A., Abdel-Daim M.M. (2022). 5-Fluorouracil-Loaded Folic-Acid-Fabricated Chitosan Nanoparticles for Site-Targeted Drug Delivery Cargo. Polymers.

[18] Radu I.-C., Zaharia C., Hudiță A., Tanasă E., Ginghină O., Marin M., Gălățeanu B., Costache M. (2021). In Vitro Interaction of Doxorubicin-Loaded Silk Sericin Nanocarriers with Mcf-7 Breast Cancer Cells Leads to DNA Damage. Polymers.

